# Serotonergic psychedelics for depression: What do we know about neurobiological mechanisms of action?

**DOI:** 10.3389/fpsyt.2022.1076459

**Published:** 2023-02-10

**Authors:** Muhammad Ishrat Husain, Nicole Ledwos, Elise Fellows, Jenna Baer, Joshua D. Rosenblat, Daniel M. Blumberger, Benoit H. Mulsant, David J. Castle

**Affiliations:** ^1^Campbell Family Mental Health Research Institute, Centre for Addiction and Mental Health, Toronto, ON, Canada; ^2^Department of Psychiatry, Temerty Faculty of Medicine, University of Toronto, Toronto, ON, Canada; ^3^Centre for Complex Interventions, Centre for Addiction and Mental Health, Toronto, ON, Canada; ^4^Mood Disorders Psychopharmacology Unit, University Health Network, Toronto, ON, Canada

**Keywords:** psychedelics, hallucinogen, depression, psilocybin, connectivity, LSD, ayahuasca, neurobiology

## Abstract

**Introduction:**

Current treatment options for major depressive disorder (MDD) have limited efficacy and are associated with adverse effects. Recent studies investigating the antidepressant effect of serotonergic psychedelics—also known as classic psychedelics—have promising preliminary results with large effect sizes. In this context, we conducted a review of the putative neurobiological underpinnings of the mechanism of antidepressant action of these drugs.

**Methods:**

A narrative review was conducted using PubMed to identify published articles evaluating the antidepressant mechanism of action of serotonergic psychedelics.

**Results:**

Serotonergic psychedelics have serotonin (5HT)2A agonist or partial agonist effects. Their rapid antidepressant effects may be mediated—in part—by their potent 5HT2A agonism, leading to rapid receptor downregulation. In addition, these psychedelics impact brain derived neurotrophic factor and immunomodulatory responses, both of which may play a role in their antidepressant effect. Several neuroimaging and neurophysiology studies evaluating mechanistic change from a network perspective can help us to further understand their mechanism of action. Some, but not all, data suggest that psychedelics may exert their effects, in part, by disrupting the activity of the default mode network, which is involved in both introspection and self-referential thinking and is over-active in MDD.

**Conclusion:**

The mechanisms of action underlying the antidepressant effect of serotonergic psychedelics remains an active area of research. Several competing theories are being evaluated and more research is needed to determine which ones are supported by the most robust evidence.

## 1. Introduction

Major depressive disorder (MDD) is a chronic and debilitating mood disorder impacting the lives of approximately 300 million people worldwide (1). First-line pharmacotherapy options include selective serotonin reuptake inhibitors (SSRIs) and serotonin and norepinephrine reuptake inhibitors (SNRIs) (2). Other antidepressants, adjunctive pharmacotherapies, psychotherapies, and brain stimulation can be used in patients who do not respond to first- or second-line treatment (2). However, 30–50% of patients do not respond to available treatments (3). Moreover, several weeks are required to determine the efficacy of pharmacotherapies and they are associated with adverse effects (e.g., weight gain, sexual dysfunction) (4, 5). Given the high prevalence of MDD, novel treatments with rapid onset are needed.

Serotonergic (“classic”) psychedelics are a broad category of drugs that includes psilocybin, dimethyltryptamine (DMT, the psychoactive ingredient of ayahuasca), and lysergic acid diethylamide (LSD). These psychedelics have been used as therapeutic agents for thousands of years in various cultures (6). Clinical research into psychedelic treatments began in the 1950s; about 40,000 individuals had been studied by the late 1960s when concerns about their safety and their recreational use led to their classification as Schedule 1 narcotics in the US (7). This precluded human studies until the late 1990s when a “second wave” of modern clinical trials restarted investigating the efficacy and safety of classic psychedelics for major depressive disorder (MDD) and other mental disorders.

Emerging evidence suggests serotonergic psychedelics have antidepressant effects (8–12). Most studies have been conducted using psilocybin for MDD, treatment-resistant depression (TRD), or end-of-life distress (11–15). Psilocybin is primarily administered in conjunction with a form of non-directive and supportive psychotherapy (11–13). Adverse effects are transient and mild, including nausea, anxiety, and minor blood pressure elevations generally resolving within the 8-h dosing session (11–13).

Despite preliminary evidence suggesting serotonergic psychedelics have rapid antidepressant effects, their mechanism of action is not well-understood. This narrative review provides an overview of their putative antidepressant mechanisms of action.

## 2. Pharmacodynamics and molecular science

### 2.1. Pharmacodynamics

Serotonin influences brain circuits responsible for regulation of mood, reactivity to stress, and cognitive performance. For more than 60 years, alterations of monoamine neurotransmitters, including serotonin, have been hypothesized to underlie depressive symptoms (16). This is indirectly supported by the fact that most traditional antidepressants impact monoamines; for example, SSRIs acutely increase the availability of serotonin in synapses (16). Additionally, depletion of tryptophan an amino acid required for 5-HT synthesis, induces depressive symptoms in previously treated patients (17, 18).

There is robust evidence supporting that the psychoactive effect of serotonergic psychedelics is mediated by their agonism of the 5-HT2A serotonin receptor (5-HT2AR). This effect can be blocked by administering 5-HT2AR antagonists, such as ketanserin or risperidone (19). 5-HT2AR agonism increases serotonin release, impacting excitability of pyramidal neurons in the cortex and inducing glutamate release in the neocortex (20). In MDD, an inverse correlation has been observed between depressive symptoms and the degree of 5-HT2AR stimulation (21). Similarly, psychedelics may exert their effects through their potent 5-HT2AR agonism leading to rapid down regulation of these receptors (22). 5-HT2ARs also play a role in cognitive inflexibility and rumination, core symptoms of depression (22). While it has been proposed that the hallucinogenic experience and associated “mystical effects” produced by 5-HT2AR agonism are responsible for the antidepressant effect (23), there is little evidence to support this. In fact, in pre-clinical models of depression, psilocybin reverses anhedonia even when mice are pre-treated with the 5-HT2AR antagonist ketanserin (24). Other receptors may be involved in psychedelics’ antidepressant effect. LSD is a partial agonist of 5-HT2AR (25, 26) and it exerts its effects mostly through agonism of 5-HT1AR (27, 28). Both LSD and SSRIs impact 5-HT1AR by desensitizing post-synaptic 5-HT1AR resulting in increased serotonin release (28). More research is needed to determine the role of specific 5-HT receptors, with each psychedelic drug requiring discrete investigations.

### 2.2. Brain derived neurotropic factor, neuroplasticity, and neurogenesis

Psychedelics’ 5-HT2AR agonism indirectly activates glutamate networks that impact prefrontal areas and downstream increases in brain derived neurotropic factor (BDNF), which supports growth and maintenance of neurons and enhances neuroplasticity. Decreased BDNF levels have been observed in MDD and correlate with suicidal behavior (29, 30). Antidepressants (31), sleep, and physical exercise increase BDNF (32, 33). Higher baseline BDNF levels correlate with higher SSRI-associated improvement in depressive symptoms (34). In rats repeated administration of ayahuasca increases hippocampal BDNF levels (35). Similarly, healthy humans and patients with TRD show increased circulating BDNF levels after a single dose of ayahuasca (36), with serum BDNF levels negatively correlated with depressive symptoms (36). Psilocybin also increases peak BDNF levels, regardless of whether patients are pre-treated with the antidepressant, escitalopram or placebo, suggesting that psilocybin has a strong impact on BDNF (37). A study using varying doses of LSD showed acute increases in BDNF only for certain doses (38), suggesting further investigation is needed. Increases in BDNF levels also play a role in neurogenesis. Depressive symptoms have been linked to insufficient neurogenesis and neurotrophic activity (39). SSRIs increase neurogenesis (40) and their behavioral effects can be blocked by its disruption (41). 5-methoxy(MeO)-DMT (42) and psilocybin (43) increase neurogenesis in rats and the study involving psilocybin suggested this might be dose-dependent (43). More research is needed to elucidate the connections among depression, neurogenesis, and psychedelics.

### 2.3. Anti-inflammation and immunomodulation

Elevated inflammatory markers signal the body is responding to stressful stimuli and attempting to return to homeostasis (28). Inflammation symptoms are similar to depressive symptoms, including fatigue, low motivation, and irritability (44). Evidence has shown that a subset of patients with MDD have increased inflammatory markers such as Interleukin 6 (IL-6) and 1β, C-reactive protein (CRP), and Tumor Necrosis Factor-alpha (TNF-α) (45, 46). Inflammatory cytokines also increase metabolism of tryptophan which may contribute to depression in some patients (47, 48). A majority of immune cells express 5-HT receptors and it has been hypothesized that serotonergic psychedelics impact immunomodulatory agents through 5-HT2AR agonism (49). Congruent with this hypothesis, several *in vitro* studies have shown decreases of IL-6 in human cells following administration of DMT (50), 5-MeO-DMT (50), LSD (51), or psilocybin (52). Similar findings are reported for other inflammatory markers such as IL-1β, CRP, and TNF-α (50, 53). Studies with ayahuasca suggest psychedelics may have immunomodulatory effects through decreasing white blood cells (CD4) and elevating natural killer cells (54). Overall, depressive symptoms have been linked to a shift of the immune system toward inflammatory responses. Although further evidence is required, serotonergic psychedelics may, in part, exert their antidepressant effect through activation of anti-inflammatory and immunomodulatory actions.

## 3. Neuroimaging

Table 1 presents a summary of the reviewed neuroimaging findings. Most studies of psychedelics have used functional magnetic resonance imaging (fMRI) with network connections assessed using functional connectivity (FC). FC measures correlations between Blood-Oxygen-Level-Dependent (BOLD) signal fluctuations (55) as a proxy for brain activity (56). Research has focused on the default mode network (DMN), encompassing the medial prefrontal cortex (mPFC), posterior cingulate cortex (PCC), precuneus, and angular gyrus. The DMN is implicated in self-referential thinking, memory, and rumination. Alterations in DMN connectivity may underlie the excessive internal focus and rumination of depression. DMN hyper-connectivity has been reported in MDD patients and correlates positively with rumination (57). Imaging data indicates that psychedelics generally reduces within-network DMN FC. This is evidenced by studies in healthy volunteers showing reductions of within-DMN FC during the acute psychedelic experience or post-treatment with psilocybin, ayahuasca, or LSD (55, 56, 58–65). Positron emission tomography (PET) scans show psilocybin increases in glucose metabolism in the PFC, anterior cingulate, and temporomedial cortex (66). In depressed patients, reductions in within-DMN FC has been observed after treatment with psilocybin (67–69), specifically between the vmPFC and right angular gyrus (69), and between the parahippocampus (PH) and PFC (67). Reduced PH-PFC FC has been associated with improvements in depression scores post-treatment (67), supporting the hypothesis that hyper-connectivity of the DMN underpins depressive symptoms. Thus, administration of a psychedelic could alleviate symptoms by reducing FC in the DMN. Indeed, increased glucose metabolism in the DMN area is positively associated with changes to perception and ego dissolution (66). Also, psychedelic-induced reduction in DMN FC has been correlated with positive changes in psychosocial functioning, attitudes and mood, 4 months post-administration (55) and with decreased mental reflection on one’s past (59).

**TABLE 1:** Summary of neuroimaging studies.

References	Pre-registration?	Treatment	Control	Sample size	Observed post-dosing effect	*P*-value	Effect size	# of studies reporting consistent findings	# of studies reporting inconsistent findings	Strengths	Limitations
Carhart-Harris et al. (67)	No	Two psilocybin sessions (10 mg D1 + 25 mg D2)	None	15	Reduced left amygdala CBF	<0.050	NA	0	0	Multiple analyses of fMRI performed (CBF, FC), correlation analysis performed (fMRI findings and treatment response), validated depression measures	Small sample size, absence of control, lack of blinding
				16	Increased within-DMN FC	<0.050	NA	1	11		
				16	Decreased within-DMN FC	<0.050	NA	11	1		
Roseman et al. (79)	Yes	Two psilocybin sessions (10 mg D1 + 25 mg D2)	None	19	Increased right amygdala responsiveness to fearful faces	0.022	NA	0	0	Preregistered, correlation analysis performed (fMRI findings and treatment response), validated depression measures	Small sample size, absence of control, lack of blinding 2-week washout possibly insufficient
					Increased right amygdala responsiveness to happy faces	0.001	NA	0	0		
Mertens et al. (69)	Yes	Two psilocybin sessions (10 mg D1 + 25 mg D2)	None	19	Increased FC between amygdala-visual areas	<0.050	NA	0	0	Preregistered, correlation analysis performed (fMRI findings and treatment response), validated depression measures	Small sample size, absence of control, lack of blinding, 2-week washout possibly insufficient
					Increased FC between DMN-visual areas	<0.050	NA	2	0		
Doss et al. (70)	Yes	Two psilocybin sessions (20 mg/70 kg D1 + 30 mg/70 kg D2)	None	19	Increased within-DMN FC	0.010	0.64*	1	11	Preregistered, correlation analysis performed (fMRI findings and treatment response), robust analyses, validated depression measures	Small sample size, absence of control, lack of blinding
				22	Increased cognitive flexibility	<0.001	0.35**	1	0		
Doss et al. (70)	Yes	Two psilocybin sessions (10 mg D1 + 25 mg D2)	None	16	Decreased brain modularity	0.012	0.72*	7	0	Placebo controlled and double-blind (DB-RTC), preregistered, validated depression measures, differences in time of fMRI scan between trials strengthens validity, robust and thorough analyses, correlation analysis performed (fMRI findings and treatment response)	Small sample size in open-label trial, inherent lack of reliability in relating psychological processes (flexibility) to brain modularity, possibility for in-scanner sleep with closed-eye fMRI
					Reduced within-DMN FC	0.009	0.75*	11	1		
					Increased between-network FC	0.010	0.72–0.75*	7	0		
		Two psilocybin sessions (25 mg D1 + D2)/2 × 1 mg psilocybin + 6 weeks escitalopram	Two × psilocybin (1 mg) + escitalopram (10–20 mg)	43	Decreased brain modularity	0.039	0.47*	7	0		
					Increased cognitive flexibility	NA	NA	1	0		

First and second doses of psilocybin are represented as D1 and D2, respectively. fMRI, functional magnetic resonance imaging; FC, functional connectivity; CBF, cerebral blood flow; DMN, default mode network. Effect sizes are expressed as Cohen’s d = (*) or as partial eta squared *η*2*P* = (**). The column titled “# of studies reporting consistent findings” refers to the number of studies reviewed which report findings that are consistent with the post-dosing effects observed in each main depression study. Similarly, the column “# of studies reporting inconsistent findings” reflects the number of studies reviewed whose findings that are inconsistent with the post-dosing effects observed in each main depression study. NA signifies information pertaining to effect sizes is unavailable.

However, increases in FC have been observed post-psilocybin in depressed patients between the vmPFC and the bilateral inferior lateral parietal cortex (iIPC) (67) and between the ACC and PCC (67, 70). Increased vmPFC-iIPC FC (but not increased ACC-PCC FC) was associated with treatment response at 5-weeks (67). It has been theorized that these discrepancies may result from differences in neural effects immediately following the acute psilocybin experience vs. those that occur later. Like electroconvulsive therapy (ECT), psychedelics may cause acute decreases in network integrity followed by a period of re-integration and subsequent improvements in mood and functioning (67). This interpretation is speculative and more research is needed to elucidate how altered connectivity patterns mediate antidepressant activity of psychedelics.

Emotional processing is also altered by psychedelics through changes in amygdala responsiveness and FC changes between the DMN, amygdala, and visual cortices. Single photon emission tomography (SPECT) in depressed patients administered ayahuasca showed increased blood perfusion in the left nucleus accumbens, right insula, and left subgenual area (9). These areas play a major role in emotional regulation and are less active in depressed patients (9). The amygdala is also hypersensitive to negative stimuli in depressed patient (71, 72) resulting in negative cognitive bias (73) and deficits in emotional regulation (74). SSRIs reduce amygdala responsiveness (75, 76) and a similar mechanism has been proposed for the antidepressant effect of psychedelics. In healthy volunteers, LSD reduce reactivity of the left amygdala to fearful faces (77) and psilocybin reduces response of the right amygdala to negative and neutral stimuli, with these reductions correlating with increases in positive affect (78). In depressed patients, opposite results have been reported: psilocybin induced increased right amygdala BOLD response to emotional faces (with a larger increase with fearful than with happy faces) 1 day post-treatment; larger responses to fearful faces correlated with higher improvements in depressive symptoms (79). This suggests psychedelics may actually restore emotional responsiveness in depressed individuals, in contrast to the emotional blunting associated with SSRIs (79). In patients with TRD, 1 day post-psilocybin, fMRI scans taken while viewing fearful faces also revealed reductions in connectivity between the vmPFC and right amygdala; it was associated with less rumination 1 week later, suggesting that disinhibition of the right amygdala by the vmPFC mediates psilocybin-induced increases in amygdala responsiveness in these depressed individuals (69). Under the same conditions, higher connectivity was also observed between the vmPFC and the right lateral occipital cortex, occipital pole, and fusiform gyrus; it was associated with improvements in depressive symptoms (69). Similar hyper-connectivity between the DMN and the occipital cortex has been reported in healthy volunteers after administration of LSD (64) or psilocybin (65), and between the amygdala and occipital cortex after administration of psilocybin (80). Deficits in down-regulation of the visual cortex in response to negative emotional stimuli has been demonstrated in MDD (81). Thus, psychedelics may help to normalize emotional processing in depressed patients by increasing vmPFC-mediated inhibition of the occipital cortex in response to negative stimuli, thereby reducing negative attentional bias. Taken together, these findings suggest a complex interplay among signals from the DMN, amygdala, and visual cortex may be involved in regulation of emotional response induced by psychedelics in MDD.

Finally, psychedelics increase connectivity between the intrinsic functional networks, i.e., spatially distinct brain regions that are functionally related (82). Brain-wide network disconnectivity is associated with depressive symptoms (83). Thus some antidepressant effects of psychedelics may also result from their ability to increase between-network FC. These increases have been observed in healthy volunteers after administration of psilocybin (56, 61, 84) or LSD (60, 62), particularly in areas rich in 5-HT2AR (60). In depression, psilocybin has been shown to increase global FC (68, 70). In one open-label trial, increase in global FC 1 day after psilocybin dosing was associated with improvement in depressive symptoms 6 months later (68). Specific increases were observed between the DMN and Executive Network (EN), Salience Network (SN). A randomized controlled trial comparing two doses of psilocybin vs. 6 weeks of daily escitalopram for depression saw increased EN dynamic flexibility with psilocybin only–reflecting an increase in the frequency of connectivity changes seen the during fMRI scan. This correlated with symptom improvement 6 weeks post-dose (68). Psilocybin has also been shown to alter connectivity patterns between the task positive network (TPN) (85), which is involved with external or other-processing (82), and the claustrum, or the DMN. Theoretically, the claustrum, known to be volumetrically reduced in depression, may mediate psychedelic-induced network disruptions due to its widespread connectivity (85).

Most psychedelic studies discussed in this section suffer from limitations, in particular difficulties in blinding and small sample sizes. Still, fMRI studies in depression have produced relatively consistent findings and their designs have been rigorous. As summarized in Table 1, of the six fMRI depression studies reviewed, five were preregistered (68–70, 79), all used validated depression scales (67–70, 79) and one was double-blinded and placebo-controlled (68).

## 4. Neurophysiology

As summarized in Table 2, neurophysiology studies provide further support for DMN-related alterations. Electroencephalogram (EEG) recordings in healthy volunteers given LSD or ayahuasca show reductions in broadband oscillatory power and cortical synchrony particularly within areas of the DMN (86–88). Similarly, magnetoencephalography (MEG) shows broadband desynchronization of cortical oscillatory rhythms and decreases in network integrity after ingestion of psilocybin (89), supporting that psychedelics can disorganize spontaneous brain activity (58, 89). In participants who ingested LSD, EEG, and MEG studies have also showed a relationship between decreased alpha power and the hallucinatory experience and subjective reports of ego dissolution (87, 90).

**TABLE 2:** Summary of neurophysiology studies.

References	Pre-registration?	Treatment	Control	Sample size	Observed post-dosing effect	*P*-value	Effect size	# of studies reporting consistent findings	# of studies reporting inconsistent findings	Strengths	Limitations
Carhart-Harris et al. (12)	No	LSD in saline (75 μg/10 mL)	10 mL saline	20	Increased CBF in visual cortex associated with decreased alpha power in OC	0.029	NA	0	0	Motion correction took place for analyses of neuroimaging data making it robust	Not performed as a double blind RCT. Performance of neuroimaging techniques at separate times.
					Decreased alpha power associated with hallucinations	<0.05	NA	2	0		
Muthukumaraswamy et al. (89)	No	Psilocybin in saline (2 mg/10 mL)	Saline	15	Reduction in oscillatory power in delta, gamma, alpha, beta, and theta bands	<0.05	NA	3	0	Robust statistical analyses of measures. Vast neuroimaging data collected	Low generalizability to population of those that have no experience using psychedelic drugs. Removal of MEG data due to head movement. Small sample size.
Murray et al. (87)	No	Two LSD sessions (13 μg and 26 μg)	Water	22	Reduction in oscillatory power in delta, gamma, alpha, beta, and theta in MPC, PCC, TPC	<0.05	NA	3	0	Blinded study with participants acting as their own controls. Robust EEG data collected	Affected brain structures were inferred from the location in which the extra cranial electrodes were located. Small sample size.
Riba et al. (86)	No	Two doses of Ayahausca (0.6 mg DMT Kg^–1^, 0.85 mg DMT Kg^–1^)	Water	18	Decreased absolute power of theta, delta, alpha, and beta	<0.05	NA	3	0	Blinding and placebo control were used while participants acted as their own controls.	Small sample size
Kometer et al. (88)	No	Placebo with ketanserin 50 mg, and placebo with psilocybin (215 μg/kg)	Placebo	17	Decrease in alpha power in POC after pretreatment with placebo	<0.01	NA	1	0	Blinding and placebo control were used while participants acted as their own controls. Validated measures used such as SCID	Small sample size

This table highlights the main studies into psilocybin-assisted therapy and neurophysiological outcomes reviewed. Brain regions are signified by their abbreviations; CBF, cerebral blood flow; PC, posterior cingulate cortex; MPC, medial prefrontal cortex; TPC, tempo parietal cortex; POC, parieto-occipital cortex; OC, occipital cortex. The column titled “*#* of studies reporting effect” refers to the number of studies reviewed which reported findings that are consistent with the post-dosing effect observed in each main neurophysiology study included in the table. Similarly, the column “*#* of studies reporting contrary” refers to the number of studies reviewed which report findings that are contradictory to the effect observed in each main neurophysiology study. NA signifies information pertaining to effect sizes is unavailable.

High frequency gamma waves appear when individuals are performing cognitively challenging tasks requiring focus (66). The limited current literature reports increases in gamma power in individuals administered psilocybin. This supports the theory that the DMN is affected by psychedelics as the PFC and hippocampus are activated during tasks requiring concentration in which gamma waves are present (66). However, given the sparsity of relevant human data, this theory remains speculative. Evidence suggests that changes from delta to gamma waves are consistent across different psychedelics (91). Additionally, LSD, ayahuasca, and psilocybin induce alpha power reductions within the occipital and parietal cortices, which are important structures for the interpretation of visual stimuli (88). It is believed this reduction explains subjective reports of visual hallucinations as alpha oscillations have been found to play a role in cortical processing of sensory information (92). Finally, under the influence of ayahuasca and LSD, psychedelic effects such as ego dissolution and spiritual feelings have been reported during moments of global decreases in alpha waves (92). Alpha oscillations are correlated with functions in which the DMN plays a major role such as self-reflection; as discussed above, fMRI studies support this association (93). Psychedelic-induced ego dissolution in patients with MDD is correlated with improvement in depressive symptoms, supporting the theory that ego dissolution is associated with decreased connectivity within the DMN and further establishing a possible link between alpha oscillations and the DMN (92, 93).

## 5. Psychology

Many depressed individuals experience ruminations, with intense self-focus and narrowed thinking. Enhanced neural and cognitive flexibility associated with psychedelics may enhance psychological flexibility, affording them access to a broader frame of mind (68). Thus, it has been hypothesized that psychedelics facilitate transitions away from maladaptive thought patterns, which can be amplified during the “integration” component of psychedelic-assisted psychotherapy (PAP) post-treatment (70). The antidepressant effect of psychedelics has also been postulated to result from their ability to restore emotional responsiveness (86), allowing people to fully experience and accept their emotions (76, 86). This emotional release is thought to be facilitated by disruption of connectivity in regions with high density of 5-HT2ARs (75). Ego dissolution, characterized by a blurring of the distinction between self and other (68) is commonly experienced with psychedelics (63, 67, 68). It has been speculated it allows for an enhanced sense of connection with others and lessen feelings of loneliness. Finally, “mystical experiences” may also play some role in relieving depressive symptoms but this has not yet been evaluated (74). In summary, psychedelics may facilitate some degree of psychological transformation, possibly allowing patients to move beyond their depression.

## 6. Discussion

Overall, available evidence suggests serotonergic psychedelics impact depression, in part through serotonin receptor agonism, neurogenesis, immunomodulation, widespread changes in connectivity within the brain, and psychological effects. To date, neuroimaging studies provide the most robust investigation into the mechanisms of action of psychedelics, but their results remain limited. More studies with larger and more diverse samples are needed to replicate and extend current findings. Future studies should also account for symptom severity and comorbidities while assessing whether and how PAP and social supports contribute to the therapeutic action of psychedelics. Neural correlates of the antidepressant effects of psychedelics and other pharmacotherapies should be compared directly to quantify differences between mechanisms of action.

Understanding the role of the psychedelic experience and its relationship with antidepressant effect should also be a focus of future research. As it stands, alterations in perception induced by psychedelics are a major barrier to their clinical adoption because they require intensive psychological support. As stated above, it is possible that psilocybin’s antidepressant effect is mediated through rapid activation of 5-HT receptors other than those involved in the psychedelic experience. This raises the possibility of blocking the psychedelic experience by co-administering 5-HT2A antagonists, like ketanserin or risperidone, without impeding the antidepressant therapeutic effect. More research is needed to explore this possibility and better understand the relationship among serotonin receptors, psychedelic effect, and antidepressant effect.

## 7. Conclusion

As use of, and research on, psychedelics expand, understanding their mechanism of action through molecular science, neuroimaging, and neurophysiology is critical. With several relevant competing theories, well-designed studies need to determine which mechanisms are central to their therapeutic action and which ones are epiphenomenal.

## Author contributions

MIH and DC conceived and designed the manuscript. MIH drafted and critically reviewed the manuscript. NL conducted the literature search, reviewed available data, and contributed to the writing, editing, and preparation of the manuscript. EF and JB contributed to the literature search, data review, and writing of the manuscript. JR, DB, BM, and DC drafted the manuscript. All authors approved the manuscript.
